# Supplementary material for: Cisplatin Induces Apoptosis in Mouse Neonatal Testes Organ Culture
Source: Int J Mol Sci. 2022 Nov 1;23(21):13360. doi: 10.3390/ijms232113360 (PMC9658841; doi:10.3390/ijms232113360)

Supplementary data (S1)

ROS-related gene expression profiling in MTFs after cisplatin treatment.

The gene expression of *Srxn1*, *Fmo2*, *Nqo1*, *Fth1*, *Gstp1*, *Scd1*, *Gpx3*, *Ptgs2*, *Prnp*, *ldh1*, *Apoe*, *Cyba*, *Gpx1*, *Gclc*, *Gpx7*, *Hmox1*, *Gss*, *Sod2*, *Txnrd1*, *Ccs*, *Psmb5*, *Gpx5*, *Nfb*, *Txn1*, *Prdx3*, *Hspa1a*, *Gpx4*, *Ctsb*, *Txnip*, *Prdx5*, *Prdx1*, *Gclm*, *Sqstm1*, *Lpo*, *Ehd2*, *Mpo*, *Gstk1*, *Gpx6*, *Prdx2*, *Cygb*, *Sod3*, *Park7*, *Txnrd2*, *Sod1*, *Aox1*, *Serpinb1b*, *Cat*, *Fancc*, *Ift172*, *Prdx4*, *Duox1*, *Nox4*, *ll19*, *Slc38a1*, *Dnm2*, *Gpx2*, *Txnrd3*, and *Als2* in cisplatin-treated MTFs were higher than in control. The expression levels of *Ercc2*, *Crcc6*, and *Recql4* were lower in cisplatin-treated MTFs than in control. Gene expression levels are presented as $\log_2$ value compared to the control value ($\log_2$ O). Quantitative analysis of each protein level. Data are shown using the mean and the standard error of the mean (n = 3). Statistical significance was set at $P < 0.05$.

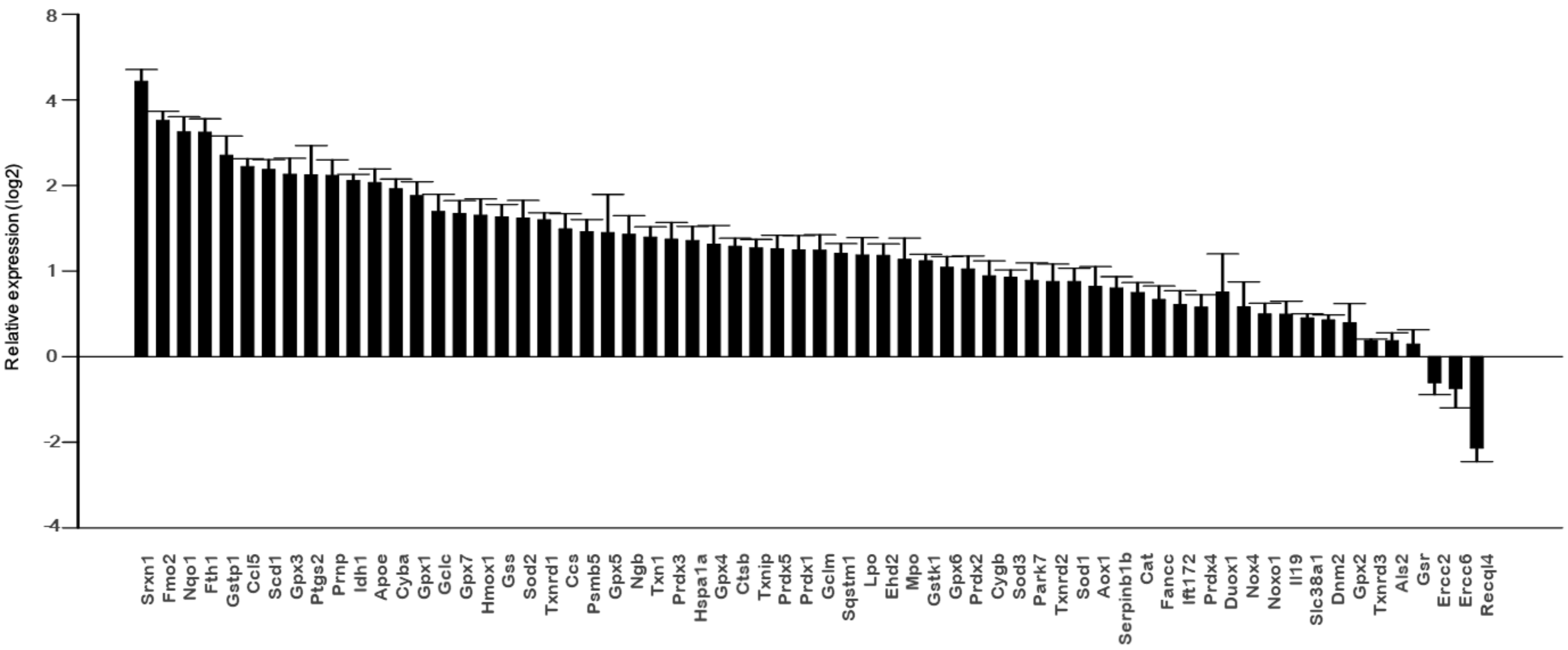

Supplement: Supplementary file 1 [file ijms-23-13360-s001.zip › ijms-1963445-supplementary.pdf]
